# Managing type 1 diabetes mellitus with a ketogenic diet

**DOI:** 10.1530/EDM-23-0008

**Published:** 2023-08-16

**Authors:** Clemens Gardemann, Sonja Knowles, Thorsten Marquardt

**Affiliations:** 1 FH Münster Oecotrophologie, Münster, Germany; 2 Clinic for Pediatrics and Adolescent Medicine/Metabolism Laboratory, Universitätsklinikum Münster, Münster, Germany

**Keywords:** Adult, Male, White, Germany, Pancreas, Diabetes, Unique/unexpected symptoms or presentations of a disease, August, 2023

## Abstract

**Summary:**

Traditional guidelines for type 1 diabetics do not restrict carbohydrates to improve clinical outcomes for patients. This paper highlights the favorable blood glucose control outcomes when a type 1 diabetic focuses on caloric intake from protein and healthy fats instead of the traditional carbohydrate-focused meals. We followed a male type 1 diabetic in his 20s adopting a ketogenic diet through a process of slowly lowering total daily carbohydrate intake. Diabetes-related biomarkers were measured throughout the process. Diabetes-related biomarkers saw massive improvements and ended up in the official non-diabetic range. Total daily insulin requirements dropped by 70%. The patient also experienced great improvements in his quality of life. This study demonstrates the possibility of improving diabetes-related biomarkers through dietary changes, which have positive effects on health outcomes in patients living with this disease.

**Learning points:**

- The adaptation of a ketogenic diet improved diabetes-related biomarkers in this patient.
- Diabetes-related biomarkers, such as HbA1c, are the main risk factors for developing complications in diabetics.
- The ketogenic diet is a feasible approach to minimizing the risk of developing complications in diabetics.
- Total daily insulin requirements dropped by 67% adapting a ketogenic diet.
- The patient experienced enormous changes in the quality of life after adapting to the new diet.
- The safe and physiological state of ketosis might be associated with additional benefits for the patient

## Background

### Introduction

Type 1 diabetes mellitus is a metabolic disease that is characterized by the destruction of pancreatic beta cells leading to inadequate insulin production. Traditional nutrition recommendations given to patients with diabetes focus on carbohydrate counting and suggest the consumption of an unrestricted diet where the low endogenous insulin production is compensated by an external insulin supply. Insulin is given as basal insulin, a delayed-release form of insulin that is needed to cover the body’s own production of glucose by the liver, a process called gluconeogenesis. In addition, bolus insulin needs to be taken with meals to cover the amount of carbohydrates and protein in that specific meal (1).

In contrast to this traditional view where the low endogenous insulin production is compensated by external insulin supply, a different view could be taken. If insulin is in short supply and carbohydrates cannot be properly metabolized, a low-carb diet would lower the amount of insulin needed. Since sugar consumption with Western diets has risen tremendously during the last 100 years (2), a low-carb diet is much closer to the diet that prevailed during most of the time of mankind.

Type 1 diabetes mellitus is a metabolic disease always caused by the autoimmune destruction of pancreatic beta cells, leading to a complete deficiency in insulin secretion (3, 4, 5). Insulin is responsible for the utilization of glucose for energy production and without it the body’s fat, liver and muscle cells cannot adequately take up this nutrient (4). Type 1 diabetes mellitus is one of the most widespread diseases occurring in childhood (4). It has a prevalence of over 85% of all diabetes cases in youths under the age of 20 years with a peak onset between the ages of 11 and 13 years (3, 4). The global incidence of Type 1 diabetes mellitus has continued to rise over the past 50 years with a rate that increases annually by about 3% (3, 4). There are several risk factors associated with the onset of this disease, including genetic and environmental factors such as the enteroviruses, exposure to cow’s milk protein, exposure to wheat gluten and lack of vitamin D. Additional risk factors include ethnicity, obesity and increased birth weight (4).

Poorly managed diabetes is characterized by hyperglycemia which carries the associated risk of long-term damage, dysfunction and failure of various organs (5). Glycated hemoglobin, also known as HbA1c, provides a standardized and reliable measure for blood glucose levels over a period of the preceding 6–12 weeks (4, 5, 6). Generated by a process called glycation, where glucose reacts non-enzymatically with hemoglobin, HbA1c levels are directly proportional to blood glucose levels; increased HbA1c levels increase the risk of diabetes-related complications and indicate uncontrolled diabetes or diabetes with hyperglycemia. The recommended target HbA1c levels for diabetes without complications is ≤6.5% (normal range: 4.0–5.6% (4, 6)).

Protein glycation due to long-term elevated glucose levels lead to the development of neuropathies, e.g. in the eyes, feet or the vagus nerve. This is not dependent on type 1 or type 2 but depends on the actual glycemic control. In a study with 117 193 non-diabetics, the mean glucose levels showed a correlation with the risk of developing irreversible organ damage. The risk ratio for a 1 mmol/L higher glucose level was 2.01 (95% confidence interval (CI): 1.18–3.41) for retinopathy, 2.15 (1.38–3.35) for neuropathy, 1.58 (1.94–2.40) for diabetic nephropathy, 0.97 (0.84–1.12) for estimated glomerular filtration rate (eGFR) <60 mL/min/1.73 m^3^, 1.19 (0.90–1.58) for peripheral arterial disease (PAD) and 1.49 (1.92–2.17) for MI (7).

Prior to the discovery of insulin, type 1 diabetes mellitus was treated with very-low carbohydrate diets (VLCD) that kept daily carbohydrate intake below 10 g (8). With the introduction of insulin therapy, the knowledge of low-carb diets in type 1 diabetes became mostly forgotten. A few studies have examined carbohydrate restrictions for patients with type 1 diabetes mellitus and reported benefits in HbA1c levels (8). Dr Richard K Bernstein, who is a type 1 diabetic himself, advocates a low-carbohydrate diet for both type 1 and type 2 diabetes. In the publication that he co-authored with Lennerz et al. (9), the glycemic control in type 1 diabetes following this dietary approach was evaluated. Reported mean HbA1c was 5.67% ± 0.66%. Only 7 (2%) respondents reported diabetes-related hospitalizations in the past year, including 4 (1%) for ketoacidosis and 2 (1%) for hypoglycemia. Exceptional glycemic control of T1DM with low rates of adverse events was reported by a community of children and adults who consume a VLCD (9). This was better than T1D patients adhering to a standard, carbohydrate-focused diet. A study with 27 035 insulin-treated T1DM children, adolescents and young adults reported a median HbA1c of 7.8% (10).

Few studies have been published addressing the benefits of a low-carb diet in diabetes. A child with type 1 diabetes came off insulin completely after strictly following a low-carb paleolithic ketogenic diet (11). A moderate carbohydrate restriction over a 12-week period from 250 to 100 greduced the time spent in hypoglycemia, glycemic variability and weight with no effect on cardiovascular risk factors (12).

An additional aspect is the relationship of carbohydrate intake to obesity. A study showed an increase in obesity in type 1s from 3.4% to 22.7%. Obesity correlates with insulin resistance, which is a predictor for the development of type 2 diabetes mellitus, emphasizing the importance of managing weight in type 1 diabetics (13).

## Case presentation

The patient presented is a German male in his 20s who was diagnosed with type 1 diabetes mellitus at the age of 2 years. He weighs 61 kg and has a height of 1.75 m. He had no known comorbidities, did not smoke and drank no alcohol at all. No other, potential metabolic risk factors were revealed during a check-up at his doctor’s office. His professional life is in the field of nutrition science. At the time of data collection, he was still studying for his bachelor’s degree. He was doing resistance training at least once a week. Since diagnosis at the age of two, the patient was on multiple daily injections, including Insulin Detemir long-acting (basal) insulin every evening and morning along with Insulin Aspart (bolus) insulin. He had a carbohydrate intake of around 140 g per day broken up into roughly 23 g at breakfast, 55 g at lunch, 12 g at snack time and 50 g at dinner. Blood glucose levels were observed and managed using the Freestyle Libre Continuous Glucose Monitor (CGM) and Freestyle Freedom Lite, HbA1c values were determined at clinical checkups with values always around 7%. The patient was one of the first in his country to acquire a CGM via insurance covered and has been using it since the very first generation. Although the patient strictly adhered to the doctor’s recommendations, including carbohydrate counting and dosing rapid-acting insulin with meals, he experienced large fluctuations in his blood glucose levels, difficulty in concentrating and thinking clearly at times, along with increased fatigue.

## Investigation

The patient prepared and managed his meals keeping track of the carbohydrate content of every meal from May 2018 to February 2019. HbA1c values were collected during routine clinical checkups every 3 months. In addition, he did an extensive lab test at the Universtitaetsklinikum Muenster, showing no nutrient deficiencies, low levels of inflammation (CRP <0.5 mg/dL) and no other impairments after following the diet for up to a year. His 25-OH-vitamin D3 level was at 44.7 ng/mL and TSH at 1.71 μU/mL. Serum calcium, magnesium and ferritin were within the reference range. Testosterone and cortisol levels were also within the reference range. In addition, the patient had to undergo the standard-of-care lab tests at the endocrinologist’s office which all diabetics have to undergo, never showing a marker out of range, thus raising anything of concern.

## Treatment

**Figure 1:**
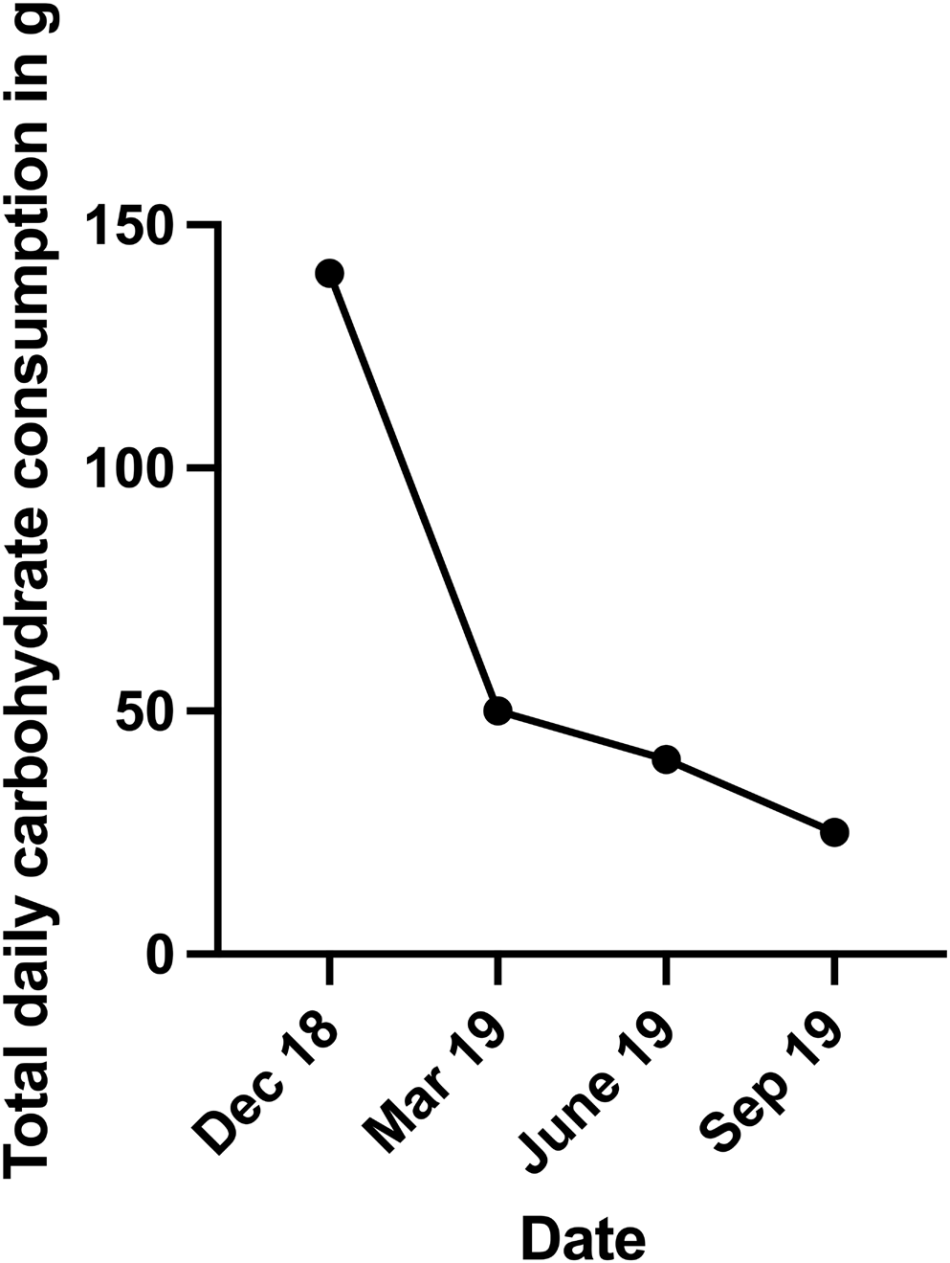
Total daily carbohydrate consumption was reduced from 140 g to 25 g within 1 year and remained the same as the patient is still following the diet till this day.

The patient began to reduce carbohydrate intake in December 2018 from approximately 140–25 g per day (Figure 1), thereby increasing his protein and fat intake to compensate for the caloric difference. He started out eliminating all sources of starchy or high-sugar carbohydrates besides carrots, while gradually lowering the total daily carbohydrate intake even further. The nutritional quality was changed during this time to include fresh vegetables, meat, eggs, goat cheese, butter and fish.

## Outcome and follow-up

**Figure 2:**
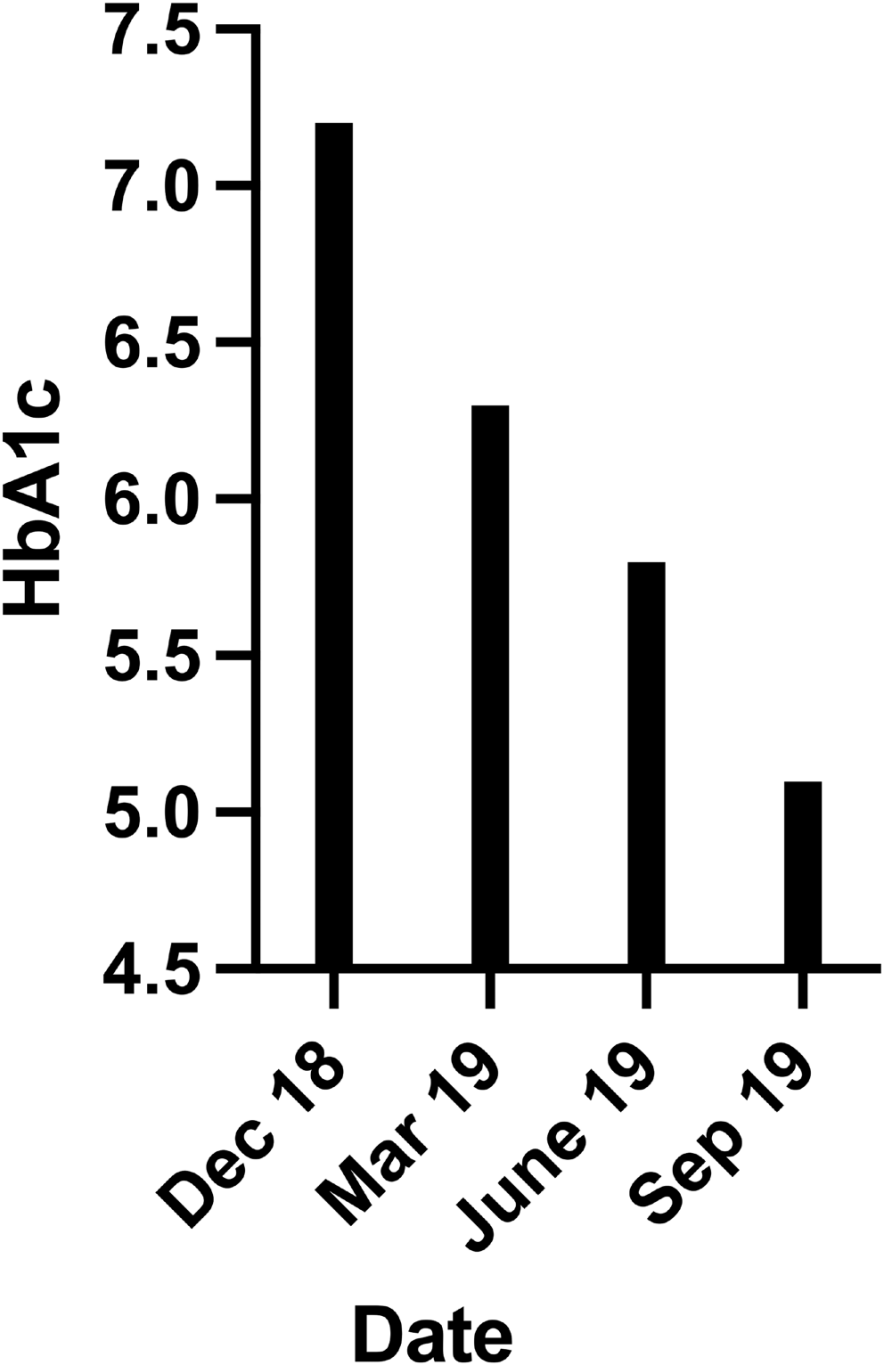
HbA1c values of the patient post-dietary change beginning at 55 mmol/mol (7.2%) in December 2018 and ending with the latest value at 32 mmol/mol (5.1%) in September 2019.

This study examined the patient over a period of 9 months. During this time the patient followed a diet where he reduced his carbohydrate intake to approximately 24–30 g per day. The dietary modification resulted in a steady and significant reduction in his HbA1c values during the duration of this study. As seen in Figure 2, the patient’s HbA1c values start at 55 mmol/mol (7.2%) in December 2018 and by the end of the study in September 2019 had dropped to 32 mmol/mol (5.1%), putting his values into the normal, nondiabetic range. HbA1c was measured in a professional clinic by the patient’s endocrinologist. Although this treatment is not a cure for the patient’s disease, reduced glycation should reduce the risks of complications associated with his diabetes.

The patient was not suffering from any major side effects besides reporting ‘carb cravings’ while starting to adjust to the diet. These started to dissipate after the first month.

No significant changes in terms of body weight or physical activity were noticed.

**Figure 3:**
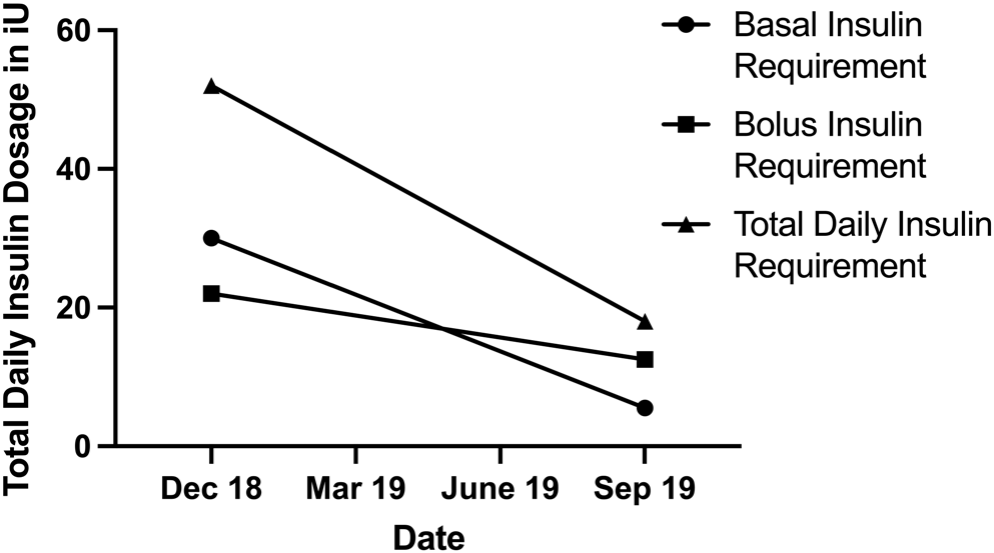
Daily amount of basal and bolus insulin changed within the timeframe of 1 year throughout the process of adapting to the new diet.

**Figure 4:**
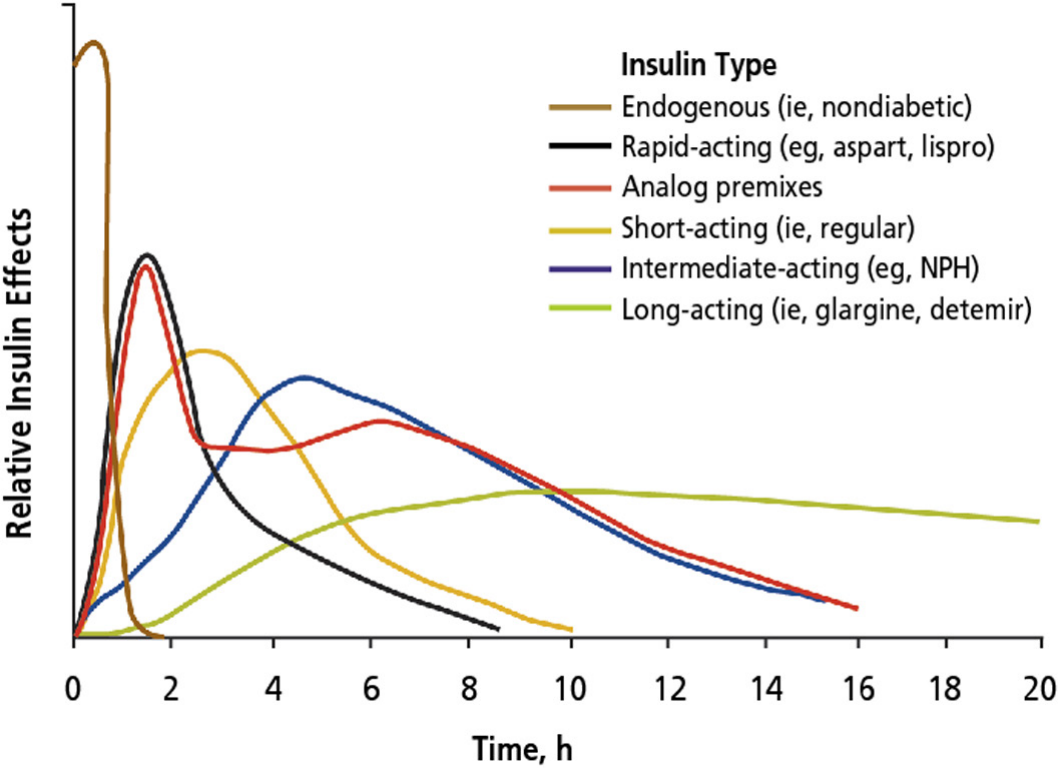
Different kinetic actions of insulin analogs compared to human insulin. This figure is reproduced with permission from Freeman (19), © 2009 The Journal of Osteopathic Medicine.

The patient’s insulin requirements changed as a result of his treatment. Before low-carb nutrition was initiated, the patient was using 30 IU of Insulin Detemir total daily and within the ongoing process of adapting to the new diet, he ended up at a total requirement of 4.5–5.5 IU of Insulin Degludec (Figure 3). Right now, Insulin Degludec is the most potent basal insulin on the market, which allows a very stable insulin absorption over a period of 18-24 hours (depending on the individual’s insulin sensitivity), so that the liver’s gluconeogenesis is covered in that timeframe. The different pharmacokinetic profiles of insulin analogs and true human insulin are shown in Figure 4. When using Insulin Detemir, the patient experienced significantly shorter action time, which increased the amount of the actual dosage and the amount of total daily shots that needed to be taken to cover the glucose produced by the liver over a period of 24 h; the patient needed to take multiple injections of Insulin Detemir a day, compared to one daily injection of Insulin Degludec. The patient observed this effect during the investigations, but we cannot state if it is specifically depended on the carbohydrate restriction or the general difference in the Insulin’s potency. Prior to the dietary modification, the patient’s bolus insulin requirements were approximately: 8 units at breakfast, 10 units at lunch, 2–4 units in the afternoon (depending on if the patient required a snack) and 8 units at dinner. Post the dietary modification, the patient’s insulin requirements, which changed from Insulin Aspart to Regular Insulin, were reduced to approximately: 2.5 units at breakfast, 5.5 units at lunch and 4.5 units at dinner. When using Insulin Aspart, the patient experienced an indifference between the actual digestion of low-carb food, such as low glycemic vegetables and proteins and the onset/peak of the insulin. The insulin was working very fast compared to the slow digestion of the food, which made glycemic control very hard. Regular Insulin, which is the actual human insulin diluted 25 fold, has a much slower onset and peak. This perfectly matches the digestion of low-carb food, thus making the glycemic control very easy to stay in a consistent range.

In case the patient’s glucose level went too high, he corrected the elevated blood glucose level with a small dosage of Regular insulin, which he injected intramuscularly into the side of his legs. In most cases, these so-called ‘corrections’ would consist of less than 1 IU of insulin. In case his blood sugar went too low (<70 mg/dL), the patient corrected the blood sugar with pure glucose tablets. Glucose tablets containing (mainly) pure glucose raise blood sugar predictably within a timeframe of 30 min in contrast to sweets containing fructose or other sweeteners, thus making precise corrections of blood glucose impossible. One gram of glucose elevates his blood glucose level by 7–8 mg/dL.

**Figure 5:**
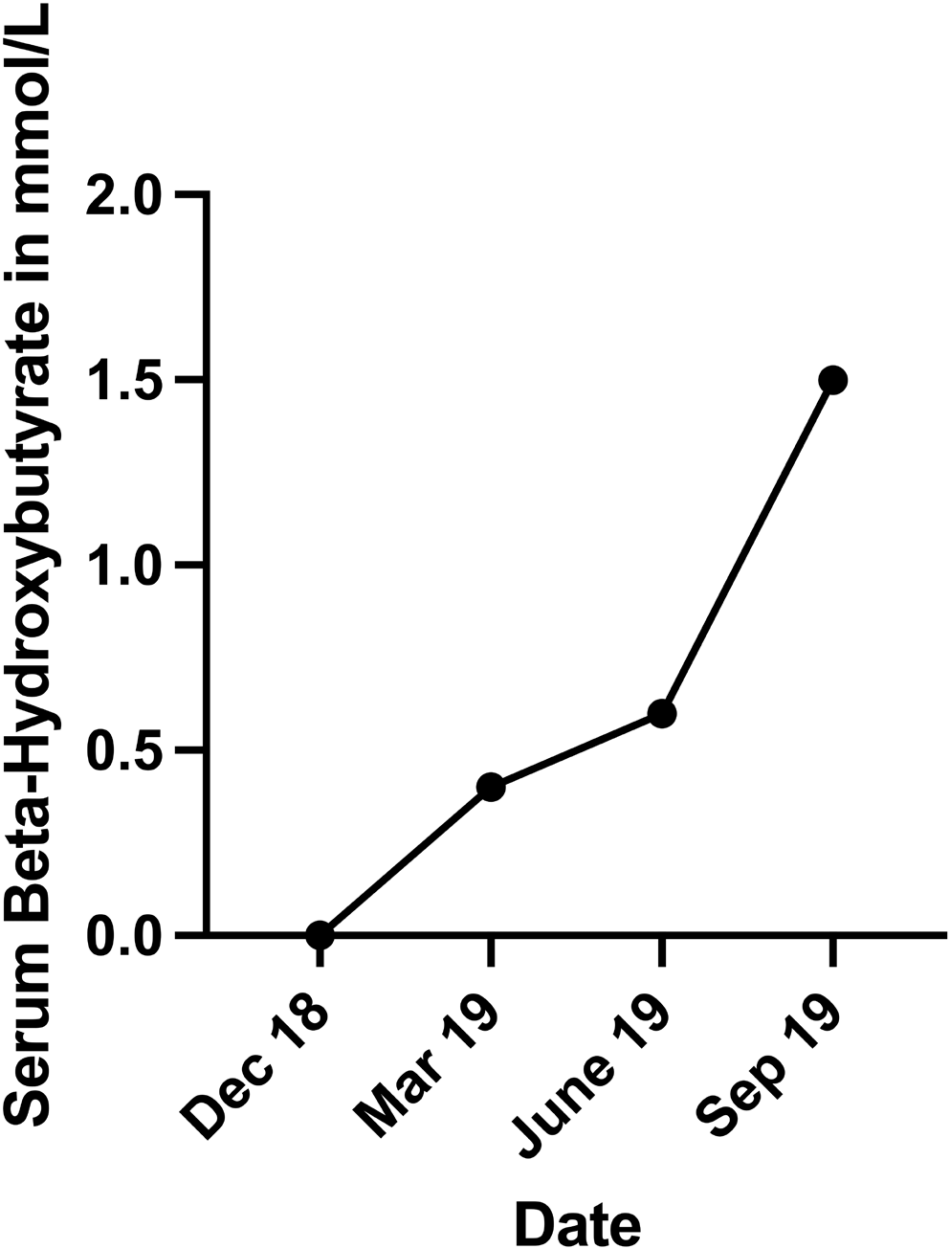
Average beta-hydroxybutyrate serum concentration while the patient adapted his new diet showing a tendency to ketosis, not to be confused with ketoacidosis.

While progressively lowering the carbohydrate content in the patient’s diet, serum beta-hydroxybutyrate concentration started to rise, achieving its average peak at around 1–1.5 mmol/L. Ketone levels varied throughout the time of day ranging from 0.2 to 2 mmol/L. Beta-hydroxybutyrate, a natural by-product of fatty acid oxidation was measured via Freestyle Beta-Ketone blood test strips. This is a safe and physiological process called ketosis, occurring in every human who fasts or follows a low-carbohydrate diet. It is an anticipated state, as ketosis is associated with potential benefits for humans (14). While it is true that diabetic ketoacidosis requires elevated levels of ketone bodies such as beta-hydroxybutyrate, those levels measured in patients diagnosed vary from 9.8 to 13.7 mmol/L while simultaneously having a blood sugar level of 476–606 mg/dL (15). Both parameters are not even remotely close to what the patient was reporting. It could even be argued that the patient is less prone to the dangers of diabetic ketoacidosis as his low glycemic variability stops blood sugar levels exceeding certain values, while diabetics following the standard approach show higher glycemic variability (Fig. 5).

During the reported timeframe, the patient did not suffer from any acute illness.

During the follow-up period, there was not a single incidence of severe hypoglycemia which has remained till today. There was also no incidence of ketoacidosis.

Arguably, the most important change seen from this treatment was the significant increase in the quality of life of the patient. Due to the extreme reduction of blood glucose fluctuations and reduced HbA1c values, the patient reported more energy, clearer thought, the ability to focus better, decreased hunger urgency and the overall feeling of greater safety and security in the management of his disease. We have to keep in mind that such a diet needs to be followed for the rest of the patient’s life in order to achieve that tight glycemic control. Transitioning to a ketogenic diet may initially appear to be limiting from the outsider’s perspective but once the dietary commitment is consistently adhered to, people report high levels of satisfaction and satiety from protein and fat-focused meals. People following a ketogenic diet need to plan their meals when eating outside of the home. The beginning of this diet seems to be especially difficult for most people as they experience carbohydrate withdrawal and tend to crave sweets and carbohydrates.

Until today, the patient is still following a low-carbohydrate diet, focusing on non-starchy vegetables, high-quality fats such as olive oil and protein sources as mentioned. Different arrangements have been made such as a lowered fat intake with a higher protein intake in conjunction with up to 30 g of carbohydrate daily (+glucose tablets), but the overall diet style remains the same.

The last HbA1c values measured were 5.2 and 5.4 mmol/L (2022/2023), while still not reporting a single incidence of severe hypoglycemia or ketoacidosis.

The patient reported discrepancies between CGM blood glucose readings and fingerstick measurements. Because of that, he started to keep track of blood glucose readings after the investigation period exclusively via the aforementioned Freestyle Freedom Lite meter. The CGM was used only to provide alarms in case the blood glucose level dropped below 70 mg/dL. At the time of his latest HbA1c value of 5.4 mmol/L (2023) his average 90-day blood glucose level was 89 mg/dL with a standard deviation of 15 mg/dL. We cannot provide data for blood glucose variability before the investigation period as the patient was not aware to take part in a case study at that time, but we could draw conclusions due to the wide difference in HbA1c levels pre- and post-intervention (7.2–5.1 mmol/L).

We focused on the documented time period as all of the professional checkups and the extensive lab test were carried out during that timeframe.

## Discussion

The findings of this study demonstrate that following a ketogenic diet can have a positive impact on patients with type 1 diabetes mellitus. This dietary modification seems to decrease blood glucose variability, decrease HbA1c levels, reduce the amount of insulin required with every meal and improve the quality of life.

Since carbohydrates are considered the main macronutrient affecting postprandial glycemic response, traditional nutrition therapy for diabetes focuses primarily on carbohydrate counting, setting a minimum of 130 g/day (7, 8, 16, 17). Emerging evidence, however, suggests that fat and protein may also have significant effects on postprandial glucose control and therefore, with further research, the complete meal composition should be considered for future insulin-dosing education (11).

Evidence from this study further brings to question whether the traditional nutrition recommendations for a high-carbohydrate diet continue to hold merit.

Though we recognize that there are limitations to this study, being that the results are primarily from the observations of a single patient, previous studies do corroborate the findings of this study. Toth and Clemens in 2015 presented the first reported case of type 1 diabetes mellitus being successfully managed with the paleolithic ketogenic diet (18). Bolla *et al.* presented reductions in HbA1c and daily insulin dose with their observed controlled study that restricted carbohydrates to 75 g/day (7). An observational study of 11 adults following the ketogenic diet (<55 g carbohydrates per day) found improved HbA1c levels and a reduction in glucose variability (7).

This shows the potential of dietary interventions in terms of low-carbohydrate diets for patients with diabetes. Future research is needed in the form of larger scale studies to confirm that the results of this case study and similar publications can be transferred to the larger population.

In summary, the management of type 1 diabetes mellitus can be improved with a ketogenic diet, a form of a VLCD . This study demonstrated the potential of such a diet to reduce HbA1c levels, reduce blood glucose variability, reduce insulin dose and improve the quality of life.

## Declaration of interest

The authors declare that there is no conflict of interest that could be perceived as prejudicing the impartiality of the research reported.

## Funding

The authors acknowledge support from the Open Access publication Fund of the University of Muenster.

## Patient consent

The patient is the leading author of this paper (C Gardemann). Written consent has been obtained from the patient after full explanation of the purpose and nature of all procedures used.

## Author contribution statement

Conceptualization: CG and SN; Methodology: CG, SJ and TM; Writing - original draft preparation: SN; Writing - review and editing: CG and TM; Supervision: TM
